# Why Are You Keeping a Brachycephalic Dog? Insights from Interviews with Brachycephalic-Dog Owners

**DOI:** 10.3390/ani16060883

**Published:** 2026-03-12

**Authors:** Judith Frehner, Sonja Hartnack

**Affiliations:** 1 Section of Veterinary Epidemiology, Vetsuisse Faculty, University of Zurich, 8057 Zurich, Switzerland; sonja.hartnack@vetmeduni.ac.at; 2 Clinical Department for Farm Animals and Food System Science, Centre for Veterinary Public Health and One Health, University of Veterinary Medicine Vienna, 1210 Vienna, Austria

**Keywords:** dog–human relationship, canine torture breeding, projection surface

## Abstract

Despite increasing efforts by the scientific community to raise awareness of breed-related health problems through educational campaigns, public information initiatives, and veterinary outreach programmes, brachycephalic dog breeds remain highly popular. As the number of brachycephalic dogs increases, the prevalence of associated health problems rises accordingly. Ethical and animal welfare considerations appear to play a limited role in breed selection. In German-speaking regions, extensive educational efforts have been undertaken in recent years to address the issue of so-called torture breeding, defined as intentional selection for extreme phenotypic traits that impair health, reduce welfare, and cause chronic suffering, particularly in brachycephalic breeds. The aim of this study was to determine the underlying reasons for the decision to buy and keep a brachycephalic dog. Although the veterinary profession is already improving education and communication, this qualitative study intended to find new starting points for targeted education against animal suffering and to explore the sociological background of the ownership of such dogs. For this purpose, semi-structured interviews with people with brachycephalic dogs were conducted throughout Switzerland (*n* = 16). The focus was on the animal–human relationship. The interviews were defined by systematically applied guidelines for the design of the interview process, while still allowing maximum openness (all possibilities for expression). The transcribed interviews were coded and analysed according to the Kuckartz methodology, which allows us to set certain focal points of analysis and to structure them according to codes. The results of this study indicate that, although awareness of torture breeding is present within the broader population, owners of brachycephalic dogs frequently rely on individualised arguments and rationalisations. These typically involve emphasising the perceived health, functionality, or exceptional characteristics of their own animal (e.g., claims that their dog is “healthy” or not affected by breed-related problems), thereby distancing their personal ownership experience from the general welfare concerns associated with the breed. This psychological pattern can be interpreted as cognitive dissonance, in which contradictory beliefs are harmonised through selective perception or re-evaluation. The results also show that brachycephalic dogs offer a very strong projection surface: their owners assign them a variety of social roles that go beyond the classic animal–human relationship—for example, as a substitute for children, a romantic partner, or a best friend. This qualitative study provides differentiated insights into the attitudes and motivations of owners of brachycephalic dogs and illustrates that traditional awareness campaigns have not been sufficient to effectively change problematic breeding practices and ownership patterns. In order to develop long-term effective solutions, interdisciplinary cooperation is therefore needed—for example, between veterinary medicine, animal welfare, communication science, psychology and law. In addition to individual education, new, target-group-specific communication strategies and consistent legal regulations are needed to protect animal welfare in the long term. This study is intended to serve as a catalyst for a broader ethical and social debate on the keeping of torture breed dogs.

## Simple Summary

The number of brachycephalic dogs is steadily increasing, regardless of scientific warnings about the health problems of this breed. Despite awareness campaigns and media reports about the suffering of the animals, the popularity of these dogs remains high. The aim of this study was to better understand the reasons for buying and keeping these dogs. For this purpose, 16 interviews were conducted with owners from German-speaking countries (southern Germany and Switzerland). The interviews showed that many owners find individual reasons to justify their dogs, although they are aware of the problems of the breed. Health benefits or the animal’s performance are often emphasised, which can indicate a kind of cognitive dissonance—i.e., the suppression of contradictory beliefs. The study provides important insights into the motivation and perception of owners. It shows that education alone is not enough to reduce animal suffering. Instead, legal measures are also needed to stop the breeding of dogs that are prone to suffering. Overall, the findings of this work call for social reflection on human preferences for pets and emphasise that animal welfare can only be achieved through a combination of education, legislation and social rethinking.

## 1. Introduction

Brachycephalic dog breeds, characterised by their uniquely shortened face and skull shape, have gained unprecedented popularity in recent decades [1]. Breeds such as the French Bulldog, Pug and English Bulldog are increasingly dominating the cityscape and social media [2]. However, this growing popularity sharply contrasts with the significant health challenges associated with extreme brachycephaly. Breathing problems, eye conditions, skin fold inflammation, and spinal abnormalities are among the consequences of torture breeding, defined as the deliberate selective breeding of animals for extreme phenotypic traits. In brachycephalic breeds, this includes extremely shortened muzzles, flattened faces, or other exaggerated features that compromise normal physiological function, impair welfare, and frequently lead to chronic pain or suffering throughout the animals’ lives [3,4,5].

Loyalty strongly influences pet owners’ choices and commitments. Studies show that many owners continue to choose and recommend certain dog breeds despite known health risks [6,7]. Emotional bonds, positive experiences, and the breeds’ distinctive traits often outweigh potential health challenges, highlighting the strength of the owner–pet relationship.

Cannas et al. [8] showed in a quantitative study that owners of brachycephalic dog breeds are aware of their health problems. This study also indicated that many owners attribute a positive health status to their animals despite their known torture breeding characteristics. Nevertheless, the question remains as to what motives and attitudes actually motivate owners to keep these breeds. It is therefore of great importance to carry out further research in order to gain a deeper understanding of the subjective perspectives of these owners. Qualitative research is particularly suitable for this, as it offers the opportunity to ask open questions and give participants space for detailed and individualised answers. This allows valuable insights to be gained into the owners’ motivations, perceptions and attitudes, which are often difficult to capture in quantitative studies.

It is argued that the physical characteristics of brachycephalic dogs—in particular their neotenous, puppy-like features (“baby schema)—provide an exceptionally large ‘projection surface’ for human relationship expectations and emotional attachments. This tendency towards anthropomorphisation, coupled with the instinctively triggered need for protection and care, appears to promote a deeper and more intense emotional bond than in normocephalic dog breeds [9]. A central aspect of the study is the extent to which this bond, which is often perceived as particularly strong, is maintained and rationalised even when the owners are aware of their animals’ health problems.

This qualitative study is based on in-depth interviews with owners of brachycephalic dogs and provides valuable, nuanced insights into their motivations, experiences and perceptions. The insights gained should not only contribute to a more precise understanding of the fascination with these breeds, but also provide concrete suggestions for the further development of communication strategies by veterinarians and animal welfare organisations.

The aim of this scientific study is to shed light on this complex motivation in detail and to conduct an in-depth exploration of the multi-layered aspects of the human–dog relationship, especially in this challenging context.

## 2. Materials and Methods

### 2.1. Approach

This research is based on a constructivist cognitive theory position, i.e., it aims to examine a particular social situation and gain a complete understanding of the experiences of others. Due to the complexity of the topic, the social science (qualitative research) approach is better suited to providing rich data than quantitative research [10].

The knowledge and perspectives of the researchers are reflected in the data and cannot be separated. This study was conducted by veterinarians for whom brachycephalic breeds and related welfare issues are a major focus of professional concern. It must therefore be taken into account that the information collected here is a result of the active research process and is not objective data [11,12].

### 2.2. Study Design

Qualitative interviews with owners of brachycephalic dogs were conducted over a period of 11 months. The owners came from German-speaking countries (southern Germany and Switzerland). Participants were recruited via word of mouth and social media forums. The recruitment criteria for inclusion in this study were that the persons must be owners of at least one brachycephalic dog (French Bulldog, Pug, Old English Bulldog), be over 18 years of age, be German speaking and agree to an audio recording of the interview.

The University of Zurich’s ethics self-assessment tool was used to check that the study was ethically sound. As the tool assessed that the project did not require any further ethical review, it was not submitted to the relevant ethics committee.

Following the principle of thematic saturation [13,14], interviews were conducted until no new themes emerged from the data. Ongoing analysis indicated that saturation was reached after 16 interviews, as additional interviews did not generate new categories but confirmed existing findings. This sample size is consistent with recommendations in the qualitative research literature for studies with a focused research question and a relatively homogeneous sample.

### 2.3. Interview Structure

The first author conducted semi-structured individual interviews with the dog owners. The focus was on the human–dog relationship. The role of the private vet was also analysed. An interview guide (File S1) with open-ended questions was developed for the qualitative study. The questions could be adapted during the interviews to follow the natural flow of conversation, allowing discussions to develop freely and ensuring thorough and diverse data collection. In addition, close collaboration with researchers from the social sciences took place as part of a peer mentoring process. Guiding questions were discussed, critically reflected upon and adapted together in order to ensure the validity and depth of the qualitative data.

The questions were not made available to the participants before the interview. The open questions allowed maximum openness (all possibilities for expression) and led to various follow-up questions and discussions. The participants were interviewed in their native language (Swiss German, German). The interviews were recorded and transcribed literally by the first author. Names of pet owners and veterinarians or practices, as well as location details, were replaced by non-identifiable descriptors (e.g., ‘veterinary practice’).

### 2.4. Data Analysis

The transcribed interviews were systematically coded and analysed using qualitative content analysis according to Kuckartz [14]. This methodological approach combines systematic structuring with interpretative analysis and allows for the focused examination of predefined and emergent aspects of the data through the development of a coding system.

Qualitative content analysis is an established method in empirical social research that aims to systematically extract, organise, and interpret information from textual material. It is particularly suited to interpretative research questions, as it enables the identification of recurring patterns, meanings, and relationships within the data while maintaining transparency and methodological rigour.

Following Kuckartz’s approach, the analysis proceeded through an iterative process. Initially, the interview material was read in full by multiple members of the research team to gain a comprehensive overview of the data. Subsequently, main categories were developed based on the research questions and the interview guide. These deductively derived categories were then supplemented by inductively generated subcategories emerging from the material itself.

Coding was conducted independently by more than one researcher, with regular discussions to compare interpretations, refine category definitions, and resolve discrepancies through consensus. The coding system was continuously revised throughout the analysis to ensure that it adequately captured the breadth and nuance of participants’ narratives.

Qualitative content analysis according to Kuckartz is based on the assumption that texts are social constructions shaped by broader social, cultural, and situational contexts. Accordingly, the analysis does not focus solely on the explicit content of statements but also considers implicit meanings, underlying assumptions, and contextual references that emerge within and between passages of text. This approach enables a nuanced interpretation of participants’ perspectives and the social meanings embedded in their accounts [13].

Qualitative content analysis according to Kuckartz is based on the assumption that the text is a social construction shaped by social and cultural conditions. The method assumes that texts consist not only of the literal statements, but also of the meanings that develop between the lines and in the context of the text [14].

## 3. Results

### 3.1. Themes and Subthemes

The final analysis with all main themes is shown in the overview in the Supplementary Materials.

The themes were classified into three main categories: (1) human traits; (2) symbiotic relationship between owner and dog; and (3) awareness.

In the following section, direct quotes from owners are printed in italics. Words or expressions in square brackets have been added to make the quotes easier to understand.

### 3.2. Theme 1: Human Traits

On analysis, it becomes evident that human traits are frequently attributed to the dogs. The character traits are described in very human terms, with hardly any that are typical for dogs. This tendency reflects anthropomorphism, the attribution of human characteristics, emotions, or intentions to non-human animals [15].

#### 3.2.1. Empathy

The dogs were described by their owners as particularly empathetic. Both empathy between dogs within the species and empathy between dogs and humans were mentioned. Empathy is the ability to empathise with the presumed emotion of another living being on the basis of cognitive understanding of this emotion while maintaining self–other differentiation.

*“She is very very very, really very empathetic. Tender but also curious. […] So basically along the lines of ‘a new dog arrives, I’m submissive first and read the situation, then I play’.”*

—*M15*

One dog owner describes how her dog goes to people in a retirement and nursing home who need closeness and even visits people who are dying.

*“[I liked the most about her, that] She has always been very empathetic. […] She is very empathetic towards people. […] In the nursing home, she always felt immediately when someone was unwell and then she deliberately lay down beside them. She also did end-of-life support.”*

—*F12*

#### 3.2.2. Affection

The description of the dogs’ affection was striking. This character trait was often mentioned first. Some dog owners described their dogs as extremely affectionate. They gave different examples of this.

*“But after all, she is very, very, very affectionate. She is very focussed on me.”*

—*F11*

*“[She is] Perfect. She is affectionate. She is of course people orientated.”*

—*F10*

*“Affection. I really appreciate that in [petname].”*

—*F2*

*“But when I’m out walking or we’re away or we’re at home, she’s always with me. So that’s when I’m in bed, when I’m on the couch, when I’m lying on the floor, she always has to be with me. Even when I’m walking, she doesn’t take a step without me. And I love that she’s like that with me. Others say it’s a sick relationship. But I think it’s great that she’s so affectionate.”*

—*F3*

*“It’s much more important for her that you lie on the sofa with her. She can lie there and wants to be stroked and cuddled. That’s her favourite thing to do.”*

—*F12*

It is also mentioned that this character trait is clearly more prominent in the participants’ dogs than in other dogs.

*“A very affectionate one. He always has to be with me or with my partner, so he’s always looking for physical contact. That’s really extreme. No other dog does that like him.”*

—*F2*

The participants were also asked which characteristic of their dog they would miss the most if it were no longer there. Affection was also mentioned here.

*“Yes, so this attachment. Because they are so affectionate. So the cuddly one. The way they always look for me. I think that’s it.”*

—*F9*

*“What would I miss? The being affectionate. The closeness. The caring.”*

—*F8*

#### 3.2.3. Stubbornness

The dog owners frequently described their dogs as stubborn, a trait that is not generally considered uniformly positive. However, owners often framed this characteristic in a loving way. Many also noted similarities or a sense of closeness between their own personalities and those of their dogs, highlighting how owners interpret and relate to their pets through a personal lens.

*“[She is] Open-minded, very empathetic. But she can also be stubborn. Very, very stubborn. Just like its human. […] We are very similar in character, it’s really funny.”*

—*F12*

*“She’s stubborn as hell. […] But yes, I’ve accepted the challenge and it’s working somehow.”*

—*F9*

Sometimes it even became a competition to see who was more stubborn. Either the dogs or the pet owners win, according to the statements made.

*“I just found this stubbornness [so cool], maybe because I’m also so insanely stubborn. I always find that funny, such a cool competition with the dog to see who can last longer. But then she usually wins.”*

—*F12*

*“He thinks he has to get his way. But I’m the bigger pigheaded one.”*

—*F8*

#### 3.2.4. ‘Baby Schema‘

Originally, the term ‘baby schema’ comes from the German ‘Kindchenschema’. It is used as a generic term for certain features, mostly facial features, that are characteristic of infants. These include a large head with a protruding forehead, large eyes, bulging cheeks and a small nose. These features play an important role in the development and survival of newborns, as they influence parental bonding [9]. Pet owners describe the facial features of their dogs according to the baby schema.

*“His eyes are of course larger than those of other dogs. They are also protruding and bulging. And his nose is small and has wrinkles.”*

—*F2*

*“She just has a short nose, little ears and cute googly eyes.”*

—*F10*

*“People always react positively to her. She just has that baby schema.”*

—*F11*

#### 3.2.5. Human Attributes

Dogs were often assigned human attributes. This was expressed, among other things, in statements that were subconsciously made as if we were talking about a person and not an animal.

*“He [the French bulldog] is a family person. [3 s break] Well I mean a family dog.”*

—*M6*

*“He [the French bulldog] always has to protect his ladies, his chicks, the two girls.”*

—*F2*

*“She has always wanted to walk everything herself since she was a baby.”*

—*F1*

During the interviews, some dog owners spoke as if they were speaking from the dog’s perspective.

*“We have never tried agility. I think she’s too lazy for that. She likes to go outdoors, but she says ‘no, I don’t fancy that sort of thing’.”*

—*F1*

*“When he hears abstract sounds from the television or something like that, he can also wake up from the deepest sleep, jump at us and say ‘what have you done?’.”*

—*F2*

### 3.3. Theme 2: Symbiotic Relationship Between Owner and Dog

There is a close relationship and dependency between owners and their dogs. This relationship can be characterised by positive qualities, such as friendship and support. Dogs are not only pets, but also loyal companions and often part of the family.

#### 3.3.1. Common Language

Communication is a very important factor in any relationship, including interspecies relationships. Some of the dog owners stated that they had even learnt a common language due to their deep relationship with their dog.

*“We have established the following: blinking once means yes and blinking twice means no. This works. Nobody believes it, but I can have conversations with [pet name] like a human. We have a connection that is beyond [good and evil]. It’s really incredible how deeply connected we are and what a relationship we have.”*

—*M15*

*“I already understand him just through his eye language. So I really understand him wordlessly. Without hand signals. really. he has such an intense expression.”*

—*M16*

#### 3.3.2. Human Replacement

With regard to human–dog relationships, some participants describe their dogs as a substitutes for humans. A distinction is made between different relationships. On the one hand, some owners describe their relationship with their dog as a friendship.

*“Sometimes I wish, without sounding as cheesy as a Lassie film, that people were as good friends to me as he is. […] He really is my best friend, like my soul mate.”*

—*M16*

*“[Pet name] and I have a really tight relationship. There’s really nothing in between. […] Not even a leaf fits between us. There’s really nothing that can get between us. […] We have a very special bond.”*

—*F12*

Dogs are perceived by some participants as more sensitive than humans, and the unspoken bond with their dog is also appreciated.

*“She simply senses it when you have a bad day and sit down. She is the first to come and sit with you. Before anyone else comes and says ‘tell me about it’. They [the other people] realise that something is wrong, but not as well as [pet name]. So it’s really also this unspoken thing. Even if they can’t talk. […] For me, dogs are often more important than people. That’s just the way it is. That’s what I often say when I’m walking through town: ‘dogs over people’. You’re walking around a lot of [strange] people, really. Then I look at [pet name] and think ‘we’ve found each other, it’s all good’.”*

—*F7*

Other owners describe their relationship with their dog as a kind of partner substitute.

*“When my husband went through a phase like that (…) he made the comment that I would rather have the dog than him anyway. Then I replied coldly ‘yes, that’s true’.”*

—*F7*

*“I’ve been single for a while now. Mostly the whole of his (the dog’s) life, so the last 8 years. But I don’t feel like I’m single because I have him. I’m not lonely. I would say I’ve seen a lot of people come and go in my life. But he is the only lasting thing, the only one I can trust blindly.”*

—*M16*

Also, some owners make it clear that their dog is a replacement for children for them.

*“She is our child, if I may say so.”*

—*M6*

*“I’ve been single more or less since then, with a few and very brief forays into the ladies’ world. I am a single pug daddy, very happy and [pet name] is my everything. […] After neutering, I was the helicopter pug daddy 24/7.”*

—*M15*

*“In fact, you could say it’s a third child. To be honest.”*

—*F5*

*“I also often say to my mother ‘The foster child is coming back’ or ‘Mum, I’ll bring my child back to you’.”*

—*F3*

#### 3.3.3. Guardian

The dogs were sometimes given the role of guardians.

*“He’s always on his toes. If someone comes along who doesn’t suit him, he feels he has to protect me. […] People’s reactions are also different, some of them are scared.”*

—*F14*

*“I would never want anything else [other than a Molosser]. This really powerful, bulky one. A dog that protects me and stands there and also represents something.”*

—*F7*

*“I prefer to have a dog that goes forwards, not a fearful one.”*

—*F5*

#### 3.3.4. Social Catalysers

Dog ownership gives people access to other human relationships. Dogs can act as social catalysers by bringing people together and encouraging interaction. They often facilitate contact between people, be it in the park or improving relationships between owners and their parents. For others, their dog enables easier access to people who are potential life partners.

*“What I personally find very touching is my relationship with my father. He is now 84 years old. I have to say that I am anything but a wanted child and therefore anything but emotional. And thanks to [dog’s name], my father has learnt to show emotions even in his old age. […] I’m currently changing jobs. I’m really well received by the people there now. Everyone is really looking forward to [dog’s name], she already has her water bowl, a dog bowl and a blanket.”*

—*M15*

*“[I particularly like] the fact that you have someone to go for a walk with.”*

—*M6*

*“We went through a really difficult time in our family. I then had the feeling that something [the dog] joyful had to come into the family.”*

—*F5*

*“When women are around, he [dog’s name] always tries to ensnare the best woman and get her on his side. And he really is a charmer.”*

—*M16*

#### 3.3.5. Not Being Alone

Together with their dog, some pet owners state that they do not feel alone.

*“I feel more comfortable and confident. And since I’ve had him [the dog], and it’s been 8 years now, I sleep better. I sleep better, I sleep more peacefully and I no longer have asthma. My asthma was of a psychosomatic nature. I am self-employed and always have a lot of stress. he grounds me.”*

—*M16*

*“It fills the empty space.”*

—*F7*

*“Instead of talking to myself, I have conversations with my pug.”*

—*M15*

*“We are always together 24 h a day. She [the dog] will leave a huge hole. She is my first dog, I don’t know what it will be like. But I always take her with me everywhere, she’s my companion.”*

—*F11*

#### 3.3.6. Being in Love

When some owners talk about their dogs, they mention the infatuation they feel for the animals. Some also talk about falling in love at first sight.

*“I took her for a walk and spent a weekend with her. And then I was hooked. It was love at first sight. The chemistry was right. She was always watching me, always looking at me as if she wanted me to tell her something. then I knew it was my dog. I then adopted her.”*

—*F11*

*“When I saw this animal [the pug] for the first time, I fell in love with the face. And the character.”*

—*M15*

*“Every dog is good-natured and super. But the Frenchies really got to me. Yes, I would never have thought that it would hit me so hard. I also decorated with Frenchies and hung up pictures. I really am just a total fan.”*

—*F9*

#### 3.3.7. Dog as the Meaning of Life

For many dog owners, their dog is the meaning of life. Keeping them satisfied is their first priority. The value of dogs is often placed above the value of humans.

*“It might be dangerous to say that. I could be accused of being a sociopath. But he [the dog] is actually my number one priority. Strictly speaking, he’s at eye level with me. […] [To give up the dog because of a move or a partner or something.] That would not be something I would accept. I would show that person the door. I’m very straightforward, there’s no right or left. He [the dog] is family. If someone doesn’t like him or has a problem, no chance. I throw him out of my life. […] I am so happy with him. It was the best choice of my life. I have never regretted it and would do it again. Even though I’ve had to do without a beach holiday for 8 years now.”*

—*M16*

*“That’s a fact. I also choose my work so that I can take the dog with me. If not, then they’ve seen the last of me.”*

—*M15*

*“Until she [dog] came along, I didn’t realise how much someone can grow fond of a dog like that. When she had to go, I sat on the floor in the kitchen and cried every day for three weeks. I really did. I didn’t go out anymore, I didn’t go for walks, I didn’t do anything.”*

—*F7*

### 3.4. Theme 3: Awareness/Denial

#### 3.4.1. Owner Does Not Perceive Any Medical or Physical Limitations in the Dog

The dog owners were asked whether there were any complications in their dogs due to their breeds. This was answered in the negative. For many pet owners, characteristics such as loud breathing noises were not perceived as complications.

*“Midsummer is not her thing. But she breathes freely. Well you can hear her breathing, but not that bad. She really doesn’t have any problems. But in the height of summer, that’s obvious. When it’s very warm, she doesn’t want to move.”*

—*F13*

*“So when it’s hot, I only go for a walk with a cooling waistcoat. And we only walk until he has peed and defecated. We don’t walk forever like other people in the city on asphalt. I take him to the forest in the air-conditioned car. Then we go for a walk with the cooling waistcoat until he’s cleaned up and then we go home again. So no I don’t have any restrictions with him.”*

—*M16*

*“I could do anything I wanted with her. So no, I don’t have any restrictions with her. […] Well she does have a lot of issues, but nothing to do with breathing. She has severe hip dysplasia, a dislocation of the patella and mild spondylosis, a chronic allergy-induced rhinitis. She also has a grade one mast cell tumor and a grade one chronic renal insufficiency.”*

—*F11*

#### 3.4.2. Breathing Freely

Pet owners stated that their dogs could breathe freely despite the brachycephalic breed. Such statements were made independently by owners of Pugs and French Bulldogs.

*“Their priority is the breeding of healthy French Bulldogs. As much as possible. They only breed dogs that have large nostrils and are breathing freely.”*

—*F9*

*“That’s why I now have [Petname] from a kennel that breeds free-breathing pugs.”*

—*F14*

*“I was just lucky that she is breathing freely.”*

—*F11*

#### 3.4.3. Sportiness

The dogs were often described as sporty.

*“I took a sporty French bulldog. She is really very, very fit. […] She is also really breathing freely, she hardly snores. And she’s petite, rather slim and, as I said, very, very sporty.”*

—*F11*

*“She is a pug, long-legged and athletic.”*

—*F3*

*“I always say I have a sports pug. She’s not incredibly long-legged, but her legs are so tall that she can run without any problems.”*

—*M15*

##### Activity Until the Point of Collapse

When asked whether their dog experienced any medical restrictions in daily life, one owner replied that her dog is extremely active, stating that the dog’s activity level was so high that it would go into overdrive.

*“When the two [dogs] play with each other, they would play until they drop. […] When other dogs are her size, she actually plays, yes. So then she actually plays until she collapses. […] I don’t do dog sports. But I do play with the ball. She loves it more than anything. You really have to stop her at some point, because otherwise she’ll play until she faints.”*

—*F4*

#### 3.4.4. Self-Interest and Animal Welfare

##### Putting Self-Interest Before Animal Welfare

During the interviews, it became clear repeatedly that the pet owners were aware of the restrictions imposed by the breeds. The dog owners often stated that their dogs had characteristics that could not be considered healthy. Nevertheless, they made a conscious decision to keep such dogs.

*“When she was little, she still had a nose. But that became less and less over time. So now she doesn’t have this super flattened face, but she doesn’t have much of a nose. That was actually important to me, I also did some research on the internet. But I really wanted a dog like this. I just love them so much. Well also in terms of character, of course.”*

—*F4*

*“Well the dog doesn’t have to be completely healthy. But it is important to me that the skeleton is good. We go for a lot of walks and it’s important to me that he can do that too. I don’t want to carry him in my backpack or put him in the bike trailer or anything like that. I really don’t want that.”*

—*F11*

*“He [my partner] has trouble with it when the dogs have difficulty breathing. [dog’s name] was just a really, really flat faced dog, it was really sunken in. That was really really bad. He then said that the next dog would be a retropug. So yay [ironic]. I can come to terms with that. I don’t think the retropugs are ugly, they’re somehow cool too.”*

—*F12*

In the German language there is the expression “Qualzucht” (torture breeding). This is well established, especially among veterinarians. One owner openly admitted that she was aware that her dog was a so-called torture breed.

*“Yes, I just really like the face. I know what it is. I always say that when I talk to someone about it. I clearly understand that it’s agony breeding. I know that.”*

—*F4*

##### Putting Animal Welfare Above Self-Interest

A single pet owner openly stated that her dog’s welfare took priority over her own interests. She had her dog euthanised due to several illnesses.

*“It was the best decision I could have made for him. That was my biggest fear, that I wouldn’t realise in time and he would have to suffer. […] Sometimes you ignore it [the signs] out of pure selfishness. Or you just look past it. Or you don’t want to see it. And for me it really was salvation. I knew it wasn’t going to be good, so I had to let him go.”*

—*F14*

#### 3.4.5. Inconsistent Reporting

The dog owners often spoke openly about the breeds’ medical restrictions. While they acknowledged that dogs of brachycephalic breeds commonly experience health problems, many stated that their own dogs did not have any limitations. In several cases, owners made contradictory statements, for example, mentioning previous surgical interventions immediately after claiming their dog had no medical issues.

*“No, she [the dog] has not been operated on. We won’t do that either. That doesn’t make any sense. I’ve already read through this, I’m also in Facebook groups. But with an operation like this, you don’t just have to make the nostrils bigger, you have to make everything bigger, including the palate and more. And I’m not going to do that to her. [dog name—second dog] doesn’t need that either, he has a nose. And with her, no, I don’t want that. She runs and jumps. She never pants. So I really don’t do that, why should I torture a dog when I can do without it? […] So she snores a lot at night, but people do that too. She’s in great shape. It’s only when it gets a bit warmer that we have to see what we can do with her. […] We got her from a kennel. They don’t do torture breeding. So that’s another big word, ‘torture breeding’. She breeds with pride.”*

—*F9*

*“I had to operate on her when she was 1.5 years old. That’s very early. Nobody told me that the soft palate can grow back. […] I wouldn’t do that again, I would only have it operated on if there was no other option. Now we are just looking for other solutions to make it as comfortable as possible for her. We go out as early or late as possible in the summer and only stay in the shade or by the water. [Interviewer: So you don’t feel that you experience any restrictions due to the breed?] No, not at all. On the contrary. You can take a dog like this anywhere and they are super easy to handle.”*

—*F14*

*“That’s the only thing [in terms of diseases] that this dog didn’t have. So I mean the soft palate operation. But at 24 degrees, you can’t take her out anymore. She stays inside in front of the fan and everything. You really can’t take her out when it’s warmer than 24 degrees.”*

—*F7*

## 4. Discussion

With the aim of gaining insights into why people keep brachycephalic dogs, an interview study with 16 owners of brachycephalic dogs was conducted and the results were analysed by content analysis. Three central themes clearly emerged during the interviews. Firstly, the attribution of human traits to the dogs; secondly the symbiotic relationship between the owner and the brachycephalic dog; and, finally, the awareness or denial of brachycephaly (torture breeding).

### 4.1. Human Traits

The dog owners were asked to describe their dogs. They focused on human traits. Many described their dogs as particularly empathetic. When asked, the owners generally stated that their dogs showed a high degree of empathy, but especially towards humans. Empathy is the emotional response that allows an individual to align their feelings with the feelings and experiences of another. According to the empathy-altruism hypothesis, empathy leads an individual to take actions to improve someone else’s welfare [16].

Previous studies analysing the ability of dogs to feel empathy are very limited. Szánthó et al. showed that owners who had an anthropomorphic attitude towards their dogs perceived their dogs as more responsive to their emotions [17]. It has also been shown that dogs react more strongly to stressed owners than to calm ones. The closer the bond between dog and owner, the more strongly the dogs react. This is seen as a sign of empathy [16]. The brachycephalic dogs in this study therefore appear to form closer bonds with their humans than other dogs. This was also confirmed by owners who kept one or more dogs of another breed in addition to the brachycephalic dog.

The owners also often stated that their dogs showed an extraordinary affection for them. The dog–human social bond has been shaped by domestication over several thousands of years. Prior studies have provided evidence for a network in the domestic dog brain that is sensitive to familiarity and emotional content within human faces [18]. Power et al. [19] identified the ideal characteristics that a dog should have. What stood out the most was affection, combined with good health [19]. The brachycephalic breeds are considered social and companion dogs. This means that their sole purpose is to be with and accompany their owner. These animals do not have a job, such as herding sheep (e.g., Border collie) or guarding (e.g., German shepherd). However, it can be assumed that the dogs have developed further due to their proximity to humans and have a higher degree of empathy as well as affection. At the same time as the character adaptations, external characteristics were also selected for. The dogs were bred according to the so-called baby schema (‘Kindchenschema’), which refers to a set of facial features (i.e., large heads and a round face, a high and protruding forehead, large eyes, and a small nose and mouth). The baby schema concept was originally proposed as a set of infantile traits with high appeal for humans, subsequently shown to elicit caretaking behaviour and to affect cuteness perception and attentional processes [20].

The statements of the dog owners clearly show that their relationships with their dogs are very close ones. Dogs are assigned human roles, such as ‘family man’. Humans have a tendency to project human characteristics, emotions and behaviours onto animals or inanimate objects. Attributing human characteristics to dogs helps to establish an emotional connection. Dogs are often close companions and family members. By ascribing human roles to their dogs, people express their affection and the importance of this relationship. This reinforces the feeling of closeness and connection. Describing dogs as having human roles or human traits can also serve as a form of communication between people. It creates shared experiences and stories that promote understanding and bonding within social groups.

### 4.2. Dog as a Projection Surface

The close relationship between humans and dogs is unique and has developed over thousands of years [21]. Over time, dogs have adapted to socialisation with humans. They show sensitivity to the emotional state of humans [22] and to social gestures [23], and even form close relationships similar to those between infants and caregivers [24].

Dogs of brachycephalic breeds seem to have a unique role in the human–dog relationship. Apparently, these breeds offer a larger ‘projection surface’ for human relationship expectations and emotional bonds than other dogs. Despite, or perhaps because of, their anatomical peculiarities, which can limit canine facial expressions, people may interpret the facial expressions of brachycephalic dogs in a way that corresponds to their own emotional needs. The large, round eyes are often perceived as particularly expressive and ‘pleading’, which can lead to strong empathy on the part of humans. This allows for a deeper emotional projection and interpretation of the dog’s moods or needs, even if these may not be explicitly communicated by the brachycephalic dog. The dog owners interviewed reflected this in the wide variety of roles they ascribe to their dogs. The dogs were ascribed roles such as ‘king’, ‘male companion’, ‘substitute for children’, ‘entertainment programme’, ‘best friend’, and ‘partner (romantic type)’. A few stated that their dog was seen as a normal dog.

### 4.3. Awareness/Denial

In recent years, intensive educational work has been carried out in German-speaking countries regarding the health problems of brachycephalic dog breeds. The veterinary profession, animal welfare organisations and the media have repeatedly drawn attention to the serious effects of torturous breeding, which manifest as respiratory distress, eye problems, skin fold inflammation and other serious illnesses [4,25]. These campaigns have obviously had an effect: this study confirms that the population is well aware of the susceptibility of brachycephalic breeds to disease.

Despite this knowledge, however, there is a remarkable phenomenon: many owners of brachycephalic dogs develop a ‘mine is different’ mentality. They find individual arguments and explanations that portray their own dog as healthier and more capable than the breed representatives that are generally considered to be ill. This behaviour can be interpreted psychologically as cognitive dissonance. Justification strategies are developed in order to reduce the unpleasant tension between the knowledge of torture breeding and the affection for one’s own possibly suffering animal.

Owners’ arguments range from emphasising a supposedly particularly responsible breeding line, to referring to ‘normal’ breathing at rest, to attributing exceptional stamina during walks or play. Even obvious signs of breathing problems, such as snoring or strained breathing, are often played down as ‘breed-related’ or ‘cute’ instead of being recognised as warning signs of suffering. The dogs’ performance is often defined by individual, subjectively perceived observations and not on the basis of objective, veterinary criteria.

The deep emotional bond with one’s own pet plays a decisive role here. It leads to rational arguments and scientific findings being relativised in favour of positive self-perception and an idealised idea of one’s own dog. This selective perception makes it difficult to fully recognise and implement the need for measures to improve the health of brachycephalic dogs, even if there is a fundamental awareness of the problem.

### 4.4. Limitations

Every scientific study has limitations that must be presented transparently in order to interpret the results correctly. The present study is no exception. The following points are crucial for categorising the findings obtained:

#### 4.4.1. Qualitative Research and Generalisability

This study is based on qualitative research, which has specific strengths but also limitations. Qualitative methods allow a deep understanding of complex issues and the recording of nuanced perspectives of the interviewees. However, they are not designed to generalise results to the population as a whole. The findings obtained here reflect the individual experiences and attitudes of the interviewees and cannot be generalised to all owners of brachycephalic dogs. Rather, they provide a sound basis for hypothesising and further quantitative studies.

#### 4.4.2. Voluntary Participation and Selection Bias

Participation in the study was voluntary, which entails a risk of selection bias. It can be assumed that individuals who actively agreed to take part in the interviews had a heightened interest in the topic of dog ownership and were more willing to engage in a reflective discussion about their own dogs. This self-selection process may have influenced the composition of the sample and the perspectives represented in the data.

As a result, the interviewed sample may not be representative of the broader group of brachycephalic-dog owners. It is conceivable that owners with lower interest in the topic, limited willingness to reflect on breeding-related issues, or more critical or ambivalent attitudes towards brachycephalic breeding practices were less inclined to participate. In particular, individuals who perceive discussions surrounding brachycephalic dogs as sensitive, controversial, or potentially judgemental may have opted out of the study.

This limitation suggests that the findings of this study may disproportionately reflect the views of engaged and communicative owners and should therefore be interpreted with caution. The results do not represent the full spectrum of opinions among brachycephalic-dog owners, but instead provide insight into the perspectives of a self-selected group willing to participate in qualitative interviews.

#### 4.4.3. Role of the Interviewer and Possible Response Bias

Participants were informed that the interviewer was a veterinarian. This positionality may have influenced the interview dynamics and the way participants framed their responses. Under the perceived authority of a medical professional, owners may have been inclined to emphasise socially desirable aspects of their dog’s health or to downplay existing problems in order to avoid criticism or moral judgement. Such dynamics are particularly relevant in the context of brachycephalic-dog ownership, where health-related issues are subject to public and professional scrutiny. Future studies could consider making the role of the interviewer more neutral or explicitly taking this into account when interpreting the data.

#### 4.4.4. Sample Size and Thematic Saturation

The number of interviews conducted was guided by the principle of thematic saturation. Interviews were analysed concurrently with data collection in order to assess the emergence of new themes. After 16 interviews, no substantially new themes or categories were identified, and subsequent interviews predominantly reinforced previously identified patterns.

Nevertheless, it must be acknowledged that saturation in qualitative research is context-dependent and influenced by factors such as sample composition, interview depth, and analytical focus. While the sample size was sufficient to address the research question within the chosen qualitative framework, it inevitably limits the breadth of perspectives captured. Additional interviews might have revealed further nuances or divergent views, particularly among less represented subgroups of brachycephalic-dog owners.

The findings of this study should therefore be interpreted as a well-substantiated but necessarily partial account of owner perspectives, rather than as an exhaustive representation of all possible viewpoints.

## 5. Conclusions

This study provides important insights into the perceptions and justification strategies of owners of brachycephalic dogs in German-speaking countries. By focusing on brachycephalic breeds, it captured relationship dynamics closely linked to breed-specific health vulnerabilities and public discourse around so-called torture breeds. However, these findings may not be directly transferable to other dog populations.

Despite public education and awareness of welfare problems, a pronounced pattern of cognitive dissonance emerged. Owners often acknowledged general health risks while emphasising the perceived robustness or wellbeing of their own dogs, a “mine is different” reasoning that reduces tension between emotional attachment and knowledge of breed-related suffering.

Further comparative studies are needed to determine whether these patterns are specific to brachycephalic dogs or occur more broadly, and to identify factors influencing attachment and responsibility regardless of breed. Educational campaigns alone appear insufficient to change owner behaviour; a more differentiated, empathetic approach to communication is required. Veterinarians can play a key role by acknowledging owners’ emotional attachment, addressing their perceptions, promoting preventive measures, and encouraging reflection on the dog’s health without confrontation. Specific training in communication and psychology can enhance this role.

While these approaches are important, they are limited by high demand for these breeds and economic interests that often overshadow awareness of animal suffering. Complementary legal measures, such as binding health standards and stricter enforcement of animal welfare regulations, are also essential. Addressing the challenges of keeping brachycephalic dogs requires an interdisciplinary approach, combining veterinary, psychological, and sociological expertise, strong legal frameworks, and targeted communication strategies to reduce suffering and improve welfare.

## Data Availability

The original contributions presented in this study are included in the article/Supplementary Material. Further inquiries can be directed to the corresponding author.

## Supplementary Materials

The following supporting information can be downloaded at: https://www.mdpi.com/article/10.3390/ani16060883/s1, File S1: Interview guide.
