# Supplementary material for: Why Are You Keeping a Brachycephalic Dog? Insights from Interviews with Brachycephalic-Dog Owners
Source: Animals (Basel). 2026 Mar 12;16(6):883. doi: 10.3390/ani16060883 (PMC13023324; doi:10.3390/ani16060883)
Supplement: Supplementary file 1 [file animals-16-00883-s001.zip › animals-4059151-supplementary.pdf]

Interview guide

Aim: To shed light on the reasons why people decide to buy certain dog breeds and what influences this decision.

Introduction

Thank you for participating in this study. It is part of my doctoral thesis at the Vetsuisse Faculty Zurich. It is about investigating the animal-human relationship. The reasons for choosing a particular breed will also be examined. There are no right and wrong answers to the questions.

The interview is recorded to facilitate evaluation. No original recordings will be published.

Introductory questions / narrative prompts

Structured in 3 blocks

1. relationship with the dog

Description of the dog; physical characteristics as well as character (does brachycephalic appear?)

- Is it the first dog? If not, what breed before and why?
- "How did it happen that you got a dog?"
- More detailed questions
    - What characteristics of your dog are important? e.g. also in comparison to others?
    - What is the role of the dog in the family? Why is the dog important in the family? What would be missing if it wasn't there?
    - How does the environment react to the dog?
    - Why did you choose breed A and not breed B (take a similar dog, not brachycephalic for example Cairn Terrier if they say they love the size, Labrador retriever if they say friendly)
    - Ask people to tell you about their dog (age, breed, known medical problems, character, vitality, social environment of the dogs (play), heat stress)
    - Differences between the keeping of brachycephalic breeds and others
    - Are there any feelings/reactions to the keeping of your dog?

2. Information gathering/relationship with vet

- Did you inform yourself about the breed in any way before buying it? If yes, through which medium?
- Description of the role of social media regarding dog breeds
- Do you regularly visit a vet? Always the same vet? How is the relationship with the vet (how do you experience vets)?
    - Only when animal is sick or also otherwise (annual) check-ups/feed purchase ect?
- Ask about frequency of contact / trust in vet

3. Future outlook

- What does the choice look like in the future?
- Are there any reasons to choose a different dog next time?

Maintenance question

- Can you describe this to me in more detail?
- What happened next?
- Can you describe this in more detail?
- If I have understood you correctly...
- Do you think that...?
- Do I understand you correctly if...?
- Good character → what is a good character for you?
- Effort dog ownership → what is a big effort for you, what is a small one?
